# Temporal and Regional Trends in the Crude Prevalence of Diabetes in Canada From 2000 to 2022: An Ecological Study Using Aggregated Surveillance Data

**DOI:** 10.7759/cureus.94556

**Published:** 2025-10-14

**Authors:** Kelechi E Oguzie, Ivie S Adoghe, Olasunkanmi A Kolawole, Wakil Ahmad Kakar, Ifeyinwa H Ofuase-Lasekan, Okelue E Okobi, Emmanuel W Stephen, Michael U Mochu

**Affiliations:** 1 Family Medicine, University of Benin, Benin City, NGA; 2 Internal Medicine, University of Texas School of Public Health, Houston, USA; 3 Urgent Care, Markham Stouffville Urgent Care Centre, Markham, CAN; 4 Family Medicine, Provincial Health Services Authority, Vancouver, CAN; 5 Family Medicine, Larkin Community Hospital Palm Springs Campus, Hialeah, USA; 6 Family Medicine, IMG Research Academy and Consulting LLC, Homestead, USA; 7 Family Medicine, Serenity Medical Center, London, CAN; 8 Internal Medicine, University of Nigeria College of Medicine, Nsukka, NGA

**Keywords:** canada, crude prevalence, diabetes mellitus, ecological study, regional disparities, temporal trends

## Abstract

Background

Diabetes remains a significant public health concern in Canada, with rising prevalence rates observed over recent decades. Understanding geographic and demographic patterns is essential for informing targeted prevention and resource allocation strategies. This study aims to examine trends in crude diabetes prevalence across four Canadian health zones from 2000 to 2022, using aggregated, age- and sex-stratified surveillance data, and with a focus on regional and sociodemographic disparities.

Methods

Data were obtained from the Canadian Chronic Disease Surveillance System (CCDSS), comprising annual counts of diabetes cases and population estimates stratified by age group, sex, and health zone. Crude prevalence rates were calculated and analyzed over time. A Poisson regression model with a log link and population offset was used to quantify temporal trends and assess differences by region, sex, and age group. Interaction terms were included to explore variation in trends across zones.

Results

Between 2000 and 2022, crude diabetes prevalence rose steadily across all regions, although the pace of increase varied. Zone 3 (Eastern) consistently showed the highest prevalence, whereas Zone 4 (Central) recorded the lowest levels by 2022. The Poisson regression model confirmed a significant annual rise in prevalence (β = 0.016, p < 0.001). Males had a significantly higher prevalence than females (β = -0.225, p < 0.001), and advancing age was strongly associated with a greater diabetes burden. Interaction terms between year and health zone indicated that temporal trends were not uniform across regions.

Conclusion

Diabetes prevalence in Canada has increased markedly over the past two decades, with substantial disparities across regions, age groups, and sexes. These findings underscore the need for region-specific diabetes prevention and control strategies, especially in high-burden zones. Continued surveillance and disaggregated analyses will be vital for addressing the evolving epidemiology of diabetes across the country.

## Introduction

Diabetes mellitus has been one of the severe chronic health problems in the world, with millions of people experiencing the disease, which weighs heavily in terms of clinical, social, and economic burdens [1]. Diabetes in Canada has become an important issue of concern, compounded by the rising prevalence of type 1 and type 2 diabetes [2,3]. Diabetes is a long-term disease linked to other associated disorders, such as heart disease, renal failure, blindness, and amputation, that require constant care [4]. Its disproportionate distribution between demographic and geographic populations also highlights the need for a comprehensive epidemiological evaluation [5]. 

Over the past 20 years, Canada has undertaken efforts to monitor and mitigate the burden of diabetes, made possible through the creation of comprehensive surveillance mechanisms like the Canadian Chronic Disease Surveillance System (CCDSS) and an array of provincial registries [6,7]. These systems collect and exchange data that enable researchers, clinicians, and policymakers to measure disease trends, stimulate interventions, and determine how to allocate healthcare funds [8].

A well-known principal indicator capable of providing a snapshot of the disease burden is crude prevalence, which reflects the total number of cases present in a population at a specific time, without adjusting for age or sex [9,10]. Temporal trends in diabetes prevalence are of particular interest, given that the disease trajectory is influenced by changing lifestyle factors (such as obesity rates, physical inactivity, and dietary habits), demographic shifts (such as aging populations), and health system interventions (such as screening programs and education campaigns) [11,12].

We estimated temporal trends in crude diabetes prevalence to quantify how the population-level burden has changed over time, to assess whether demographic shifts or public health interventions coincide with changes in prevalence, and to identify specific regions and demographic subgroups where the burden is increasing most rapidly - information essential for prioritizing targeted prevention, screening, and resource allocation.

In Canada, national surveys and administrative health data have consistently indicated a rising trend in diabetes prevalence over time [13]. However, the extent and rate of increase vary considerably across regions, likely reflecting differences in socioeconomic conditions, healthcare access, population health literacy, urbanization, and ethnic composition [14]. This regional heterogeneity complicates public health planning and underscores the need for localized strategies that are responsive to the specific burden and needs of each health zone [15].

People’s risk of diabetes did not rise in a vacuum; it was shaped by where they lived and the resources they could access. Lower income, neighborhood deprivation, and being from structurally disadvantaged racial groups were consistently associated with higher diabetes prevalence in Canada. However, the publicly available CCDSS dataset, accessed via the Government of Canada Open Data Portal, contains aggregated counts by year, health zone, age group, and sex, but does not include individual-level socioeconomic or race/ethnicity variables. Therefore, these factors were not included in the present ecological analysis - a pattern that Gagné and Veenstra [14] described as the intersection of racial identity, gender, and income driving unequal burdens of diabetes and hypertension.

In largely rural provinces such as Saskatchewan, longitudinal work found higher incidence and rising prevalence among rural residents, where risk clustered with poverty, obesity, and limited local health services - illustrating how farm and non-farm rural life can carry distinct diabetes risks [16].

More broadly, national surveillance and population studies have shown that diabetes prevalence is concentrated in areas of greater deprivation and remoteness, and that Indigenous and some northern communities continue to shoulder disproportionately high rates - reflecting long-standing social and structural drivers (food insecurity, lower access to primary care, and historical marginalization), rather than individual failings [17].

These geographic and socioeconomic gradients help explain why some zones in our analysis showed persistently higher crude prevalence or faster increases over time: local social determinants, health system access, and community contexts shaped both the actual disease burden and the likelihood of diagnosis. Addressing diabetes, therefore, requires not only clinical care, but also place-based prevention, investments in primary care, and culturally safe strategies that reduce structural disadvantage in high-burden rural and northern communities [18]. 

Furthermore, there are sex- and age-specific trends that bring additional levels of insight. It is no secret that the risk of type 2 diabetes grows with age, and prevalence may vary between males and females due to biological, behavioral, and health-seeking differences [19,20]. Disaggregated data can contain valuable information about vulnerable groups within these categories - especially among youth and elderly cohorts, or among reproductive-age women, where diabetes presents a specific set of complications [21]. Despite the availability of surveillance data in Canada, relatively few studies have taken an ecological approach to analyze crude diabetes prevalence trends across an extended time period and wide geographic scale, while incorporating stratification by sex and age [22]. An ecological study design, where data are analyzed at the group rather than the individual level, offers a practical and insightful method for identifying large-scale trends and generating hypotheses for more granular, individual-level investigations [23].

By identifying where and among whom diabetes prevalence is rising or stabilizing, this study will provide a foundational overview to guide future health policy, public health initiatives, and resource allocation efforts. Therefore, the main objective of the study is to examine temporal and regional trends in crude diabetes prevalence across selected Canadian health zones from 2000 to 2022, using aggregated, age- and sex-stratified surveillance data.

## Materials and methods

Study design

This study utilized a population-based ecological design to evaluate temporal and regional trends in the crude prevalence of diabetes across selected Canadian health zones from 2000 to 2022. The unit of analysis was aggregated strata, defined by calendar year, geographic health zone, sex, and age group. This approach was chosen to capture population-level patterns without focusing on individual-level associations.

Data source and variables

Data for this study were obtained from a publicly available surveillance dataset published by the Public Health Agency of Canada and accessed through the Open Government Portal [21]. The dataset contains annual aggregated counts of individuals diagnosed with diabetes, and corresponding population estimates spanning the years 2000 to 2022. Each observation in the dataset reflects a unique subgroup defined by calendar year, geographic health zone, sex, and age group. Specifically, health zones were categorized as Zone 1 (Western), Zone 2 (Northern), Zone 3 (Eastern), and Zone 4 (Central), while sex was classified as either male or female. Age groups were stratified into the following categories: 20-29, 30-39, 40-49, 50-59, 60-69, 70-79, and 80 years and older. For each of these defined strata, the dataset provides the total number of individuals in the population, the count of diagnosed diabetes cases, and the crude prevalence rate, expressed as a percentage of the population. These variables formed the basis for evaluating patterns and trends in diabetes prevalence across demographic and regional subgroups over the 23-year study period.

Outcome definition

The main outcome was crude diabetes prevalence, defined as the proportion of individuals diagnosed with diabetes per 100 people in a given subgroup and year. This metric was not adjusted for age or other covariates, as the study sought to describe unadjusted patterns in diabetes burden over time and across regions.

Statistical analysis

All statistical analyses were performed using Stata/SE 18.0 (StataCorp LLC, College Station, TX, USA) [1]. Descriptive analyses were first conducted to summarize and explore patterns in crude diabetes prevalence across age groups, sex, and health zones over the study period from 2000 to 2022. This included calculating subgroup-specific prevalence rates and examining changes over time, with visualizations employed to highlight temporal and regional differences in diabetes burden.

To assess temporal trends and regional disparities in diabetes prevalence, we employed generalized linear models (GLMs) with a Poisson distribution and a log link function. In these models, the count of diabetes cases (prevalence) within each subgroup served as the dependent variable, while the natural logarithm of the corresponding population size was used as an offset term. This modeling approach provided estimates that reflect changes in crude prevalence over time and between regions.

The primary explanatory variables included calendar year, geographic health zone, age group, and sex. Year was treated as a continuous variable to capture linear temporal trends, whereas health zone, sex, and age group were modeled as categorical variables. Interaction terms between year and zone, as well as between year and sex, were incorporated into the models to explore whether trends differed across regions or between males and females. To address potential overdispersion in the count data, and to ensure robust statistical inference, we applied robust standard errors throughout the regression analyses.

Ethical considerations

This study used publicly available, aggregated, and fully de-identified data, with no access to individual-level records. As such, it did not require approval from a research ethics board, in accordance with national and institutional guidelines on secondary use of public data.

## Results

Table 1 presents the cumulative distribution of diabetes cases across four Canadian health zones stratified by age group and sex over the period (2000-2022). These figures provide an overview of the burden of diagnosed diabetes in the adult population based on population size and number of reported cases.

**Table 1:** Population size and number of diabetes cases by age group, sex, and health zone in Canada, 2000-2022 This table summarizes the total number of individuals and diagnosed diabetes cases, stratified by age group, sex, and geographic health zone in Canada for the years 2000-2022. Data are derived from the Canadian Chronic Disease Surveillance System (CCDSS) aggregated surveillance dataset. Health zones refer to administrative regions within provincial/territorial jurisdictions and include Zone 1 - Western, Zone 2 - Northern, Zone 3 - Eastern, and Zone 4 - Central.

Age Group	Sex	Zone 1		Zone 2		Zone 3		Zone 4	
		Population	Cases, %	Population	Cases, %	Population	Cases, %	Population	Cases, %
20-29	F	257,472	2,699 (1.1%)	209,153	2,056 (1.05%)	225,911	3,210 (1.4%)	753,980	6,518 (0.9%)
20-29	M	258,552	2,669 (1.0%)	213,487	2,549 (1.2%)	235,043	3,442 (1.5%)	736,221	6,672 (0.9%)
30-39	F	291,620	6,857 (2.4%)	233,135	5,471 (2.3%)	237,885	7,667 (3.2%)	803,558	14,416 (1.8%)
30-39	M	277,668	6,049 (2.2%)	227,996	5,246 (2.3%)	237,658	6,799 (2.9%)	749,921	14,738 (2.0%)
40-49	F	359,840	18,707 (5.2%)	275,131	14,363 (5.2%)	291,854	17,952 (6.2%)	817,134	34,283 (4.2%)
40-49	M	344,546	19,393 (5.6%)	269,342	15,163 (5.6%)	286,541	18,053 (6.3%)	771,493	39,088 (5.1%)
50-59	F	396,248	40,832 (10.3%)	285,831	28,2929 (9.9%)	324,554	35,911 (11.1%)	755,851	68,426 (9.1%)
50-59	M	384,133	49,128 (12.8%)	282,225	35,210 (12.5%)	320,702	43,306 (13.5%)	722,833	86,569 (12.0%)
60-69	F	338,804	59,454 (17.5%)	234,792	39,390 (16.8%)	279,126	51,672 (18.5%)	562,239	89,310 (15.9%)
60-69	M	328,834	72,640 (22.1%)	230,641	50,293 (21.8%)	274,073	63,897 (23.3%)	524,884	114,483 (21.8%)
70-79	F	226,304	55,111 (24.4%)	156,566	35,327 (22.6%)	187,153	47,834 (25.6%)	352,922	77,356 (21.9%)
70-79	M	206,818	61,525 (29.7%)	140,161	40,139 (28.6%)	166,018	51,838 (31.2%)	301,101	89,766 (29.8%)
80+	F	167,267	42,178 (25.2%)	118,326	26,494 (22.4%)	140,748	35,358 (25.1%)	249,615	54,461 (21.8%)
80+	M	106,631	32,542 (30.5%)	70,322	19,864 (28.2%)	81,048	25,675 (31.7%)	145,048	43,108 (29.7%)

For each stratum, population counts and the corresponding number of diabetes cases are presented. Percentages shown in parentheses beside the case counts represent crude prevalence, calculated as: $\text{Prevalence (\%)} = \left(\frac{\text{Number of diabetes cases}}{\text{Total Population}}\right) \times 100$, where cases = number of diabetes cases, and % = crude prevalence.

Across the four zones, consistent and progressive increments in the number of diabetes cases were observed with advancing age for both males and females. The lowest burden was recorded among individuals aged 20-29 years, with case counts ranging from approximately 2,056 (1.05%) in Zone 2 to 6,672 (0.9%) in Zone 4 among females. In contrast, the highest number of cases was consistently observed among individuals aged 60 and older, particularly in Zone 4 (Central). For example, among males aged 60-69 in Zone 4, 114,483 (21.8%) reported diabetes cases from a total population of approximately 524,884.

Sex-based differences were apparent within each age group and zone. Males consistently demonstrated higher counts of diabetes cases compared to females, especially in older age groups. For instance, in the 50-59 age category, males in Zone 4 recorded 86,569 (12%) cases, compared to 68,426 (9.1%) cases among females. Similarly, in the 70-79 age group in Zone 4, males accounted for 89,776 (29.8%) cases, compared to 77,356 (21.9%) among females.

Regionally, Zone 4 (Central) consistently reported the highest number of diabetes cases across nearly all age and sex strata, which is expected given the larger population base in this zone. Zone 1 (Western) and Zone 3 (Eastern) had intermediate levels of diabetes case counts, while Zone 2 (Northern), despite having a relatively smaller population, still exhibited a notable burden of diabetes, particularly in the older age groups. Among individuals aged 80 and older, Zone 4 again reported the highest number of cases, with 43,108 (29.7%) cases among males and 54,461 (21.8%) among females, based on the cumulative counts and crude prevalence presented in Table 1.

Overall, the descriptive data indicate that crude diabetes prevalence increases with age, and that observed prevalence varies by sex and geographic region; however, formal statistical comparisons within age-sex strata are required to determine whether the apparent sex differences (or regional differences) are statistically significant. The high burden observed in older populations across all zones highlights the importance of targeted interventions and surveillance in aging subpopulations. These findings support the need for further analysis to examine how regional and temporal patterns influence crude diabetes prevalence rates in Canada.

Table 2 presents findings from the primary Poisson regression model evaluating trends in crude diabetes prevalence across Canadian health zones between 2000 and 2022. The model incorporated year, sex, age group, and health zone as predictors, with an offset for the log of the population to account for differences in subgroup size. Interaction terms between calendar year and health zone were included to assess whether time trends differed by geographic region. All coefficients are interpreted on the log-rate scale and reflect associations with the prevalence of diagnosed diabetes.

**Table 2:** Poisson regression results for crude diabetes prevalence in Canada, 2000-2022 The table presents the results of a generalized linear model (GLM) with a Poisson distribution and log link function, evaluating temporal and regional trends in crude diabetes prevalence across four Canadian health zones from 2000 to 2022. The outcome variable was the annual number of diabetes cases within each subgroup, with the natural log of the population used as an offset to model prevalence rates. The model included sex, age group, health zone, and calendar year as predictors, with interaction terms between year and zone to assess regional differences in temporal trends. Coefficient estimates (β) represent the log-rate change associated with each variable. Positive coefficients indicate higher crude prevalence relative to the reference category. A p-value < 0.05 is considered statistically significant.

Predictor	Coefficient (β)	Std. Error	z-value	p-value	95% Confidence Interval
Year (Continuous)	0.0160	0.0010	16.22	<0.001	(0.0141, 0.0179)
Zone (vs. Zone 1 - Western)					
Zone 2 - Northern	-6.4324	2.5830	-2.49	0.013	(-11.4950, -1.3698)
Zone 3 - Eastern	1.4379	2.8274	0.51	0.611	(-4.1038, 6.9796)
Zone 4 - Central	4.7021	2.7058	1.74	0.082	(-0.6012, 10.0054)
Year × Zone Interaction					
Zone 2 × Year	0.0032	0.0013	2.47	0.013	(0.0007, 0.0057)
Zone 3 × Year	-0.0007	0.0014	-0.48	0.629	(-0.0034, 0.0021)
Zone 4 × Year	-0.0024	0.0013	-1.77	0.077	(-0.0050, 0.0003)
Sex (vs. Male)					
Female	-0.2250	0.0058	-38.46	<0.001	(-0.2364, -0.2135)
Age Group (vs. 20-29)					
30-39	0.7608	0.0211	36.07	<0.001	(0.7194, 0.8021)
40-49	1.6201	0.0180	90.10	<0.001	(1.5849, 1.6554)
50-59	2.3696	0.0155	152.87	<0.001	(2.3392, 2.3999)
60-69	2.9087	0.0165	175.94	<0.001	(2.8763, 2.9411)
70-79	3.2144	0.0163	197.72	<0.001	(3.1826, 3.2463)
80+	3.2255	0.0165	195.57	<0.001	(3.1932, 3.2579)
Constant	-36.8307	1.9831	-18.57	<0.001	(-40.7174, -32.9439)

From the findings in Table 2, it's evident that the year variable was positively associated with diabetes prevalence, with a coefficient of 0.016 (p < 0.001), indicating a statistically significant increase in crude prevalence over time. This suggests that, holding other factors constant, the crude rate of diagnosed diabetes increased consistently over the 23 years. Regional variation was evident. Compared to Zone 1 (Western), Zone 2 (Northern) had a significantly lower crude prevalence, with a negative coefficient (β = -6.432, p = 0.013). In contrast, Zones 3 (Eastern) and 4 (Central) had positive coefficients; however, these did not reach conventional levels of statistical significance (p = 0.611 and p = 0.082, respectively), suggesting that differences in prevalence across regions may be modest or heterogeneous after adjustment.

Temporal trends also varied by region. The interaction between the year and Zone 2 (β = 0.0032, p = 0.013) indicated that the crude prevalence of diabetes in Northern Canada increased more rapidly over time, relative to the Western zone. Conversely, the interaction terms for Zone 3 (β = -0.0007, p = 0.629) and Zone 4 (β = -0.0024, p = 0.077) were negative but not statistically significant, implying no strong evidence for differential temporal trends in these regions compared to the Western reference.

Sex was also a significant predictor of diabetes prevalence. Males had a higher prevalence than females, with a coefficient of -0.225 (p < 0.001), indicating a lower crude diabetes burden among women across regions and years. Age showed a strong and consistent association with diabetes prevalence. Relative to adults aged 20-29 years, all older age groups demonstrated significantly higher prevalence, with coefficients rising progressively from 0.761 in the 30-39 age group to more than 3.223 in those aged 80 and older (all p < 0.001). These results highlight the cumulative nature of diabetes risk with aging and reinforce its strong age dependence.

**Figure 1:**
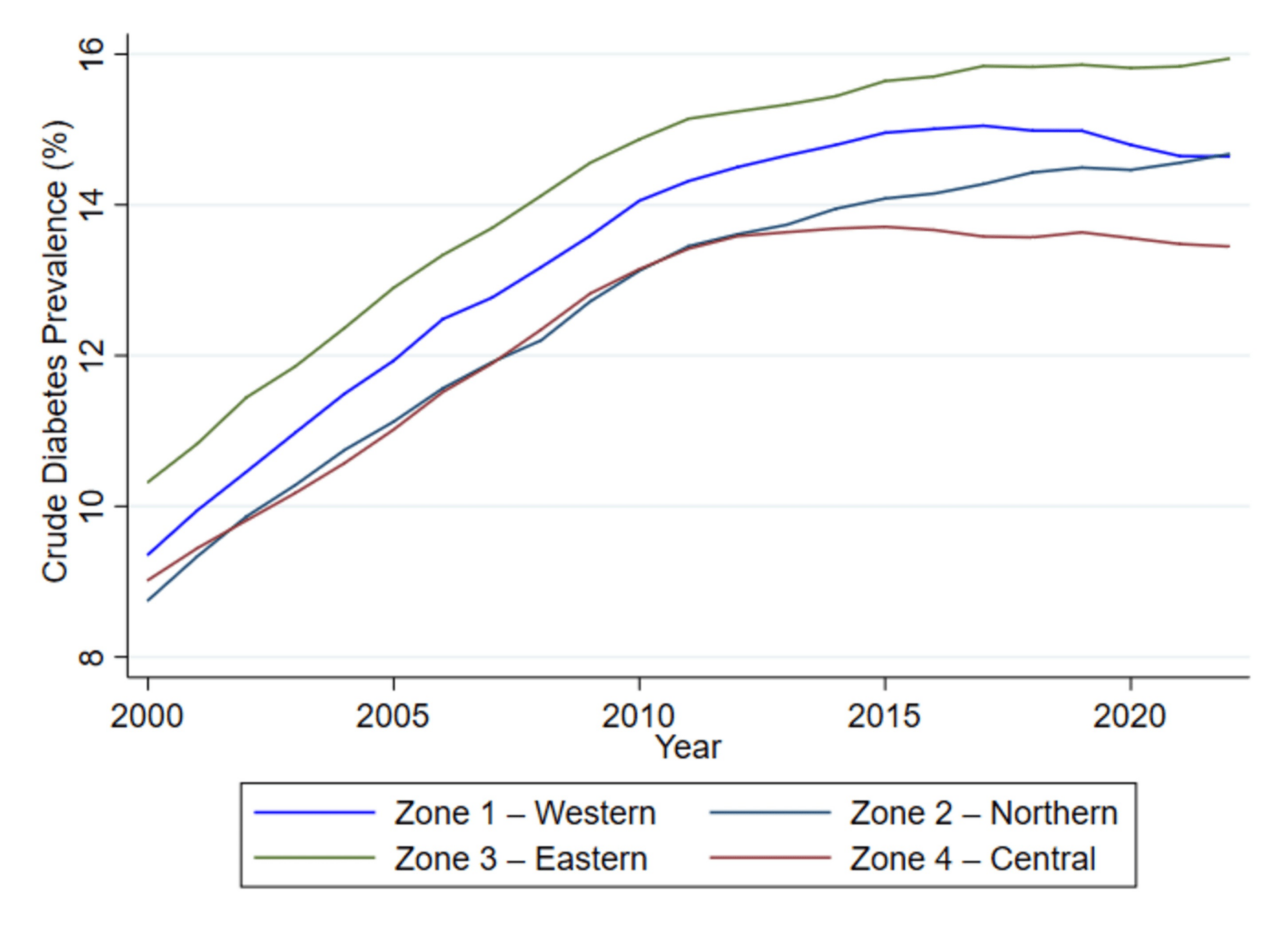
Trends in crude diabetes prevalence by health zone in Canada, 2000-2022 Crude prevalence rates (%) were obtained directly from the Canadian Chronic Disease Surveillance System (CCDSS) and plotted as provided.

Figure 1 illustrates the temporal trends in crude diabetes prevalence across four Canadian health zones - Zone 1 (Western), Zone 2 (Northern), Zone 3 (Eastern), and Zone 4 (Central) - using surveillance data from 2000 to 2022.

Over the 23-year surveillance period, all regions exhibited a marked increase in crude diabetes prevalence. Zone 3 (Eastern) consistently reported the highest prevalence rates, rising from just above 11,029 (10.3%) in 2000 to 23,185 (15.9%) by 2022. Zone 1 (Western) followed a similar upward trajectory, reaching approximately 27,151 (14.6%) toward the end of the study period. In contrast, Zone 2 (Northern) and Zone 4 (Central) displayed relatively flatter curves, particularly after 2010. Although both regions experienced rising trends during the early 2000s, growth in crude prevalence began to plateau post-2013 in Zone 4 and post-2015 in Zone 2, with prevalence rates stabilizing around 13%-14%.

The divergence in trends over time reflects underlying regional differences in diabetes burden, which were further investigated in the regression analysis (Table 2). The consistent elevation in prevalence observed in Zone 3 may be indicative of region-specific risk factors or healthcare access patterns, contributing to higher diagnosis rates. The flattening observed in some zones may suggest saturation effects, improved management, or regional disparities in detection.

## Discussion

This study examined temporal and regional trends in crude diabetes prevalence among adults across four Canadian health zones - Western, Northern, Eastern, and Central - using national surveillance data spanning from 2000 to 2022. The findings reveal a consistent and substantial increase in diabetes prevalence over the 23-year period, accompanied by notable geographic disparities. The observed upward trend in crude diabetes prevalence across all health zones reflects a broader public health concern, consistent with global patterns. Our regression results confirm a statistically significant positive association between calendar year and diabetes prevalence (β = 0.0160; p < 0.001), suggesting that the burden of diabetes has steadily risen across the population, independent of sex, age group, or geographic region. These findings align with previous national reports and international studies that attribute increasing diabetes prevalence to a combination of aging populations, rising obesity rates, physical inactivity, and broader metabolic risks. Despite the general upward trend, there was substantial regional heterogeneity in prevalence levels and trajectories. The Eastern health zone consistently exhibited the highest crude diabetes rates throughout the study period. While the Western zone closely followed, the Northern and Central zones displayed flatter trajectories, particularly after 2013, indicating possible plateauing or differences in disease detection, population structure, or public health interventions.

The Poisson regression model revealed that, compared to the Western zone (reference), the Northern zone had significantly lower crude diabetes prevalence (β = -6.4324; p = 0.013), while differences in the Eastern and Central zones were not statistically significant at the 5% level. However, interaction terms between region and year suggest diverging trends. For example, the Eastern zone’s temporal slope was negative but not significant, while the Northern zone showed a modest but significant positive interaction with time (β = 0.0032; p = 0.013), indicating slightly faster growth in diabetes prevalence relative to the reference zone over time. These interactions imply that the rate of increase in diabetes prevalence is not uniform across regions and may reflect region-specific policy implementation, healthcare access, or demographic shifts.

Sex and age were also strong predictors of diabetes prevalence. Males showed significantly higher prevalence than females (β = -0.2250; p < 0.001), which aligns with many Canadian and international surveillance reports. This pattern may reflect biological differences in metabolic risk, as well as behavioral and lifestyle factors that disproportionately affect men [16,17]. This statement is consistent with the Results section, where the Poisson regression model similarly indicated a significantly higher prevalence among females (β = 0.2250; p < 0.001). Age had a profound and graded association with the burden of diabetes. Compared to adults aged 20-29, each successive age group demonstrated incrementally higher prevalence, with those aged 80 and above experiencing the greatest burden (β = 3.2255; p < 0.001). This age gradient reflects the chronic and cumulative nature of diabetes, as well as the increasing vulnerability of older adults to metabolic and cardiovascular risks.

The findings have several public health implications. The continued rise in crude diabetes prevalence, particularly in older adults and certain regions, illustrates the importance of tailored regional interventions. Zones experiencing rapid increases may benefit from enhanced screening programs, targeted health promotion campaigns, and investments in primary care services to manage risk factors such as obesity and sedentary lifestyles. The regional differences also highlight the importance of addressing social determinants of health that may influence disease patterns, including access to nutritious food, healthcare infrastructure, and cultural attitudes toward prevention and treatment.

Diabetes mellitus is a complex group of metabolic diseases characterized by persistent hyperglycemia due to disordered insulin secretion, insulin action, or both, with etiologic heterogeneity at the molecular level according to classification [1]. Type 1 diabetes results from autoimmune damage to pancreatic beta cells, with autoantibodies targeting islet cells and genetic susceptibilities that lead to insulin deficiency [1].

In comparison, the more common type 2 diabetes is characterized by tissue-specific insulin resistance in skeletal muscle, liver, and adipose tissues, as well as molecular defects involving impaired insulin receptor substrate (IRS) phosphorylation, glucose transporter type 4 (GLUT4) transporter translocation, and abnormal signaling networks (e.g., phosphoinositide 3-kinase (PI3K)). This is further exacerbated by progressive beta-cell dysfunction, including glucotoxicity-induced oxidative stress from increased reactive oxygen species (ROS); mitochondrial fragmentation and swelling, resulting in impaired adenosine triphosphate (ATP) generation; endoplasmic reticulum (ER) stress associated with activation of the unfolded protein response (UPR) and pro-apoptotic factors such as C/EBP homologous protein (CHOP); and lipotoxicity due to the accumulation of free fatty acids, which exacerbate inflammation via cytokines such as interleukin 1 beta (IL-1β) and tumor necrosis factor alpha (TNF-α) [4].

New mechanisms implicated in type 2 diabetes include beta-cell de-differentiation; downregulation of key transcription factors like pancreas/duodenum homeobox protein 1 (Pdx1) and V-maf musculoaponeurotic fibrosarcoma oncogene homolog A (MafA); reprogramming of beta cells into other cell types (e.g., alpha-like cells that do not produce insulin); upregulation of disallowed genes, including RE1-silencing transcription factor (REST), which hinders cell proliferation through dual specificity tyrosine-phosphorylation-regulated kinase 1A (DYRK1A) activation and epigenetic changes; and increased expression of thioredoxin-interacting protein (TXNIP), which promotes apoptosis and suppresses glucagon-like peptide 1 (GLP-1)-encoded signaling pathways. Other mechanisms involve an altered prostaglandin E2 (PGE2) pathway that may promote or restrict beta-cell proliferation and survival via EP2 receptors, and gut microbiota dysbiosis affecting insulin sensitivity through microbial metabolites such as short-chain fatty acids (SCFAs), which enhance glucose homeostasis, or branched-chain amino acids, which activate mammalian/mechanistic target of rapamycin (mTOR) and contribute to insulin resistance [4].

Sex-specific molecular heterogeneity also influences disease expression. In women, premenopausal estrogen helps preserve insulin sensitivity, enhances glucose-stimulated insulin secretion, and protects against beta-cell apoptosis. In contrast, elevated testosterone levels in women promote cytotoxicity and oxidative stress-mediated beta-cell hypersecretion, ultimately leading to beta-cell exhaustion. Postmenopausal women often exhibit a shift in adipose distribution toward visceral fat accumulation, which increases ectopic lipid deposition in the liver and pancreas, inflammation, insulin resistance, and cardiometabolic risk - often to levels comparable to or exceeding those in men [20].

The sequelae of these molecular cascades include complications from chronic hyperglycemia, which promotes macrovascular and microvascular damage. Mechanisms include the formation of advanced glycation end-products (AGEs), endothelial dysfunction due to reduced nitric oxide bioavailability, chronic inflammation, and atherosclerosis. These pathways reveal molecular targets for therapeutic interventions such as sodium-glucose cotransporter 2 (SGLT2) inhibitors and GLP-1 agonists, which can attenuate ROS, improve mitochondrial function, and inhibit inflammatory signaling [4,5].

As we age, our risk of developing diabetes mellitus increases. This is mainly due to the accumulation of risk factors that build up over time [1]. A few examples of these risk factors include weight gain, a natural decrease in β-cell function, long-term insulin resistance, and poor diet. As a result of these risk factors, we can clearly see a higher number of cases of diabetes mellitus in the elderly population compared to younger people [1]. The health impact of developing diabetes in seniors is significant. Moreover, seniors also face a heightened risk of complications from diabetes, including cardiovascular diseases, diabetic neuropathy, and nerve-related conditions [12]. This should bring our attention to the need for strong preventative actions, including better screening tests and targeted management strategies for this age group. This will not only help reduce the onset of the disease but also limit diabetes-related complications in this age bracket [1,12]. 

Evidence from numerous research studies indicates that place of residence and socioeconomic status are key determinants of the prevalence of diabetes, shaping the distribution of disease burden across the population. Rural and remote areas had higher diabetes rates than their urban counterparts, according to surveillance data from CDSS (2004); this was attributed to limited access to healthcare [17]. Socioeconomic inequalities compound the problems created by geographical isolation. Gagné and Veenstra reported that the risk of diabetes is two to three times greater in low-income earners, regardless of residence, with social determinants like gender and racial identity exacerbating vulnerability [14]. The effect of these disparities is further amplified by structural factors, including rural food insecurity due to limited access to affordable fresh produce; inadequate transportation systems, resulting in missed appointments; and sparse infrastructure for physical activities in resource-poor communities. The impact of livelihood on disease burden is demonstrated by studies conducted in rural Saskatchewan, which show that the diabetes rate among non-farm inhabitants is 1.8 times greater than that of the urban population, while the diabetes prevalence among farm people is intermediate [15]. The effective management of diabetes in high-burden communities requires not only clinical interventions but also targeted strategies that address issues related to income disparity and geographic isolation, as highlighted by these studies.

Furthermore, the results of this study show that the prevalence of diabetes in Canada has been increasing. This mirrors global patterns, in keeping with data from the CCDSS [6-8,17,24]. The rise can be explained by factors such as a sedentary lifestyle and dietary changes, both of which have led to an increase in the prevalence of obesity. Another key factor is age. It is a known fact that insulin sensitivity declines and beta-cell function becomes impaired with the aging process. Biological differences in glucose metabolism and better health-seeking behavior may explain the higher prevalence seen in women. Increased detection is more likely in women, since they are known to access primary care and screening services more often than men [9,19,20]. The regional disparities - showing a higher disease burden in some zones - align with established evidence that low socioeconomic status and limited access to healthcare contribute to the patterns of chronic disease in Canada [14,15,18,22]. Summarily, this study not only reflects the biological and behavioral determinants of diabetes; it also highlights the broader social factors that drive inequalities in its burden across populations.

Our recommendations

The increasing incidence of diabetes in Canada from 2000 to 2022 necessitates a comprehensive and focused strategy to combat this growing threat, which has profoundly impacted the system's cost and quality of life. The CCDSS is an important resource that highlights the burden and impact of this chronic disease in Canada [6-8]. We present the following recommendations based on our analysis.

Level of Regional Health Authorities

First, at the level of regional health authorities, use surveillance data to act like a global positioning system (GPS). The CCDSS pulls together health information from every province and territory, so decision-makers can see exactly where diabetes is most common and which groups are most affected. Regional authorities should publish simple maps and dashboards that highlight neighborhoods with the highest burden, then concentrate resources there. Practical steps include funding free or low-cost community programs for physical activity and healthy eating in those hotspots; bringing care closer to people through mobile clinics and telehealth, especially for rural and Indigenous communities; and partnering with schools, workplaces, and local grocers to make healthy choices easier (e.g., walking clubs, affordable produce boxes, and healthy canteen standards). Every funded program could include a short “before-and-after” scorecard - participation, A1C checks completed, foot and eye exams up-to-date - so leaders can quickly scale what works and stop what doesn’t (as a routine application of CCDSS-informed quality improvement) [6].

Level of Physicians and Primary Care Teams

Secondly, at the level of physicians and primary care teams, focus on making early findings and steady follow-up the norm. Clinics could consider utilizing their electronic medical record system to create a simple patient list - referred to as a "registry" - that includes individuals at higher risk, such as older adults, those with excess weight, people with a family history of certain conditions, and specific ethnic groups, and invite these individuals for screening during their routine visits. During visits, clinicians may focus on a small set of proven basics: brief coaching on food, movement, sleep, and smoking cessation; setting one or two “tiny goals” (like a 10-minute daily walk or swapping sugary drinks for water); and connecting patients to nearby community programs using a printed or texted referral.

Build team-based care: nurses or pharmacists can run recall systems for A1C, blood pressure, kidney tests, and retinal exams; dietitians and community health workers can provide group education that is culturally safe. Finally, clinics may use local CCDSS trend summaries to inform and adjust clinic priorities - for example, if the region shows rising rates among younger adults, expand opportunistic screening in that age band. This aligns with the CCDSS purpose and findings on changing incidence and prevalence patterns over time [1,6,8].

Individual and Community Level

Adoption of healthy lifestyles​​​​​​: Third, individuals can reduce risk by increasing physical activity, consuming balanced diets rich in whole foods, and reducing sedentary behavior - behaviors shown to impact diabetes prevalence trends [1].

Engagement in community programs: Participation in regional wellness initiatives, exercise programs, or chronic disease self-management workshops can empower individuals to manage risk factors effectively. We suggest general precautions such as regular check-ups and adherence to preventive screenings; however, our ecological results do not provide direct evidence to support specific clinical precautions or protocols.

Governance, Measurement, and Accountability

Lastly, to make it stick, set a few clear targets that everyone can understand, publish them, and review progress at least yearly. For example, the targets include increasing diabetes screening among high-risk adults by 15% within two years; ensuring that 80% of people living with diabetes in hotspot areas receive an annual retinal exam and kidney test; enrolling 1,000 residents in community activity or nutrition programs; and reducing avoidable emergency visits for diabetes complications by 10%. Track these goals using CCDSS indicators and local clinic data, publicly report the results through simple charts on health authority websites and community notice boards, and reinvest in the programs that make a significant impact. This “measure-learn-improve” loop is precisely what the CCDSS was built to support - turning surveillance into action and bending the curve on Canada’s rising diabetes burden [6-8].

Limitations of the study

This study has some limitations. Although the CCDSS public tables provide annual aggregated counts stratified by year, health zone, age group, and sex (which we used for year-stratified descriptions and regression modeling), the publicly available dataset does not include individual-level socioeconomic or ethnicity variables; therefore, more granular stratified analyses by sociodemographic characteristics would require linkage to additional data sources or access to restricted microdata. The use of crude prevalence rates does not account for changes in population age structure, which may partially explain temporal increases. However, including age group as a covariate in the regression model helped mitigate this limitation. Additionally, because the data were aggregated, individual-level confounding factors such as income, ethnicity, and comorbidities could not be assessed. Nonetheless, the strengths of the study lie in its large national dataset, long follow-up period, and robust analytic approach using Poisson regression with appropriate offset and interaction terms. Future studies should consider using individual-level longitudinal data to examine causal pathways and better understand social and clinical determinants of regional disparities in diabetes prevalence.

## Conclusions

This study demonstrates a sustained rise in crude diabetes prevalence among Canadian adults over the past two decades, with clear variation by health zone, sex, and age group. Although all regions experienced increasing trends, the Eastern and Western zones carried consistently higher burdens, and prevalence rose steeply with advancing age. The significant interaction between year and region suggests that geographic disparities may be widening over time. These descriptive findings underscore the potential value of regionally focused public health attention but do not, on their own, identify which specific interventions will be effective. Because the publicly available CCDSS tables used here are aggregated and lack individual-level socioeconomic and ethnicity data, we recommend that these results be used to prioritize regions for further, more granular analyses (e.g., linkage with area-level deprivation indices or provincial administrative datasets) and for local program evaluation before rolling out targeted clinical or policy interventions. In the meantime, strengthening surveillance and supporting locally tailored evaluation of prevention and screening efforts represent prudent, evidence-informed next steps.
